# Arboviral screening of invasive *Aedes* species in northeastern Turkey: West Nile virus circulation and detection of insect-only viruses

**DOI:** 10.1371/journal.pntd.0007334

**Published:** 2019-05-06

**Authors:** Mustafa M. Akıner, Murat Öztürk, Aykut Buğra Başer, Filiz Günay, Sabri Hacıoğlu, Annika Brinkmann, Nergis Emanet, Bülent Alten, Aykut Özkul, Andreas Nitsche, Yvonne-Marie Linton, Koray Ergünay

**Affiliations:** 1 Recep Tayyip Erdogan University, Faculty of Arts and Sciences, Department of Biology, Rize, TURKEY; 2 Hacettepe University; Faculty of Sciences, Department of Biology, Division of Ecology, Ankara, TURKEY; 3 Ankara University; Faculty of Veterinary Medicine, Department of Virology, Ankara, TURKEY; 4 Robert Koch Institute; Center for Biological Threats and Special Pathogens 1 (ZBS-1), Berlin, GERMANY; 5 Hacettepe University; Faculty of Medicine, Department of Medical Microbiology, Virology Unit, Ankara, TURKEY; 6 Department of Entomology, National Museum of Natural History, Smithsonian Institution, Washington, United States of America; 7 Walter Reed Biosystematics Unit, Smithsonian Institution Museum Support Center, Suitland, United States of America; Faculty of Science, Mahidol University, THAILAND

## Abstract

**Background:**

The recent reports of *Aedes aegypti* and *Ae*. *albopictus* populations in Turkey, in parallel with the territorial expansion identified in several surrounding countries, have raised concerns about the establishment and re-establishment of these invasive *Aedes* mosquitoes in Turkey. This cross-sectional study was performed to detect *Aedes aegypti* and *Ae*. *albopictus* in regions of recent incursions, and screen for viral pathogens known to be transmitted elsewhere by these species.

**Methodology:**

Mosquitoes were collected at several locations in Artvin, Rize and Trabzon provinces of the Black Sea region during 2016–2017, identified morphologically, pooled and analyzed via generic or specific nucleic acid amplification assays. Viruses in positive pools were identified by product sequencing, cell culture inoculation and next generation sequencing (NGS) in selected specimens.

**Principal findings:**

The study group comprised 791 specimens. *Aedes albopictus* was the most abundant species in all locations (89.6%), followed by *Ae*. *aegypti* (7.8%) and *Culex pipiens* (2.5%). Mosquitoes were screened for viruses in 65 pools where fifteen (23.1%) were reactive. The infecting strains was identified as West Nile virus (WNV) in 5 pools (7.7%) with *Ae*. *albopictus* or *Cx*. *pipiens* mosquitoes. The obtained WNV sequences phylogenetically grouped with local and global lineage 1 clade 1a viruses. In 4 (6.2%) and 6 (9.2%) pools, respectively, cell fusing agent virus (CFAV) and *Aedes* flavivirus (AEFV) sequences were characterized. NGS provided a near-complete AEFV genome in a pool of *Ae*. *albopictus*. The strain is provisionally called “AEFV-Turkey”, and functional analysis of the genome revealed several conserved motifs and regions associated with virus replication. Merida-like virus Turkey (MERDLVT), a recently-described novel rhabdovirus, was also co-detected in a *Cx*. *pipiens* pool also positive for WNV.

**Conclusions/Significance:**

Invasive *Aedes* mosquitoes are established in certain locations of northeastern Turkey. Herein we conclusively show the role of these species in WNV circulation in the region. Biosurveillance is imperative to monitor the spread of these species further into Asia Minor and to detect possible introduction of pathogens.

## Author summary

Mosquitoes can transmit viruses to susceptible humans during blood-feeding. The presence and establishment of particular mosquito species within a region is the prerequisite for the introduction and emergence of the diseases transmitted by that species. *Aedes* mosquitoes transmit dengue and yellow fever, as well as recently-emergent chikungunya and Zika viruses to susceptible humans. Mosquitoes were collected in the Black Sea region of Anatolia, NE Turkey, where invasive *Aedes* mosquitoes have recently encroached, and specimens were screened for a variety of viruses. We observed particular *Aedes* species that are associated with disease transmission, suggesting that these species have been established in the region. We did not detect dengue, yellow fever, chikungunya or Zika viruses, but West Nile virus was found in several pools of these invasive species. Moreover, we detected a number of related viruses that exclusively infect mosquitoes, identified for the first time in Anatolia. Using advanced sequencing technologies, the near-complete genome of a new *Aedes* flavivirus (AEFV-Turkey) was achieved.

## Introduction

Infections due to mosquito-borne viruses have become a global health problem during the past two decades, due to their wide geographic spread and high human disease burden. This is directly associated with their widespread distribution and ecological changes related to vector mosquitoes as well as increases in international trade and travel [1,2]. Any list of the mosquito-borne viruses with significant impact that have emerged or re-emerged would include dengue virus (DENV), yellow fever virus (YFV), chikungunya virus (CHIKV) and recently, Zika virus (ZIKV) [2,3]. These viruses are transmitted to susceptible vertebrates with varying degrees of vector competence via the globally invasive *Aedes (Stegomyia) aegypti* (L.) and *Ae*. (*Stg*.) *albopictus* (Skuse) [4]. In Europe, *Ae*. *albopictus* is the most prolific invasive mosquito species, having greatly expanded its range across many countries since its original introduction to Albania in 1979 [4,5]. Despite a more constrained geographical distribution, *Ae*. *aegypti* has significantly recolonized parts of southern and southeastern Europe with populations found in Portugal and the Black Sea coast of Russia, Republic of Georgia and, most recently, in northeastern Turkey [4,5]. Risk mapping efforts have identified 215 countries or territories to be potentially suitable for the survival and establishment of invasive *Aedes* species, with disease outbreaks from more than half of the target regions [6]. Therefore, concerns of virus transmission that could initiate and sustain epidemics in countries infested by these mosquitoes have been raised, requiring vector and pathogen surveillance [3,4].

In Turkey, *Ae*. *albopictus* was initially recorded in eastern Thrace (Edirne province, bordering Greece) in 2011 [7], along with detections from several Balkan countries including Bulgaria and Romania [8]. Following DDT-based eradication efforts in the 1950s, *Aedes aegypti* was rarely identified around the Mediterranean basin, with only sporadic later reports from Turkey, Italy and Israel [8]. However, both these invasive species were recently identified at several locations from the coastal Black Sea region of Turkey, with anthropophagic adults and immature aquatic stages in used tyres stored outdoors [9]. Established populations of both species are present in neighboring Republic of Georgia suggesting local encroachment as the source of the Turkish populations. *Aedes aegypti* populations from various regions around the Black Sea demonstrate a high genetic differentiation and are hypothesized to represent expansions from remnant populations within the area [10]. Thus, a resurgence or reintroduction of viruses vectored by these species must be considered in NE Turkey, along with appropriate strategies for routine vector biosurveillance and control. Among mosquito-borne viral pathogens, West Nile virus (WNV) seems to be ubiquitous in Anatolia and Thrace regions, with several reported cases of human and equine infections [11–14]. Moreover, vector screening efforts have identified WNV in both *Culex* and *Aedes* mosquitoes in various regions [14–16]. So far, no human infections due to indigenous transmission of DENV, YFV, CHIKV or ZIKV have been documented in Turkey. However, serological evidence of sporadic exposure to DENV or an antigenically-related flavivirus [17], as well as imported cases of DENV, CHIKV and ZIKV have been reported [18–20]. In this study, we aimed to detect newly established populations of invasive mosquito species including *Ae*. *aegypti* and *Ae*. *albopictus* in regions of the Black Sea coast and screen them for viral pathogens known to be transmitted by these species.

## Methods

### Ethics statement

The study involved testing of field-collected mosquitoes for which no local or institutional ethics committee approval is required. Peridomestic collections were undertaken with informed consent and cooperation of the property owners, householders or local authorities.

### Study area, specimen collection and identification

**Fig 1:**
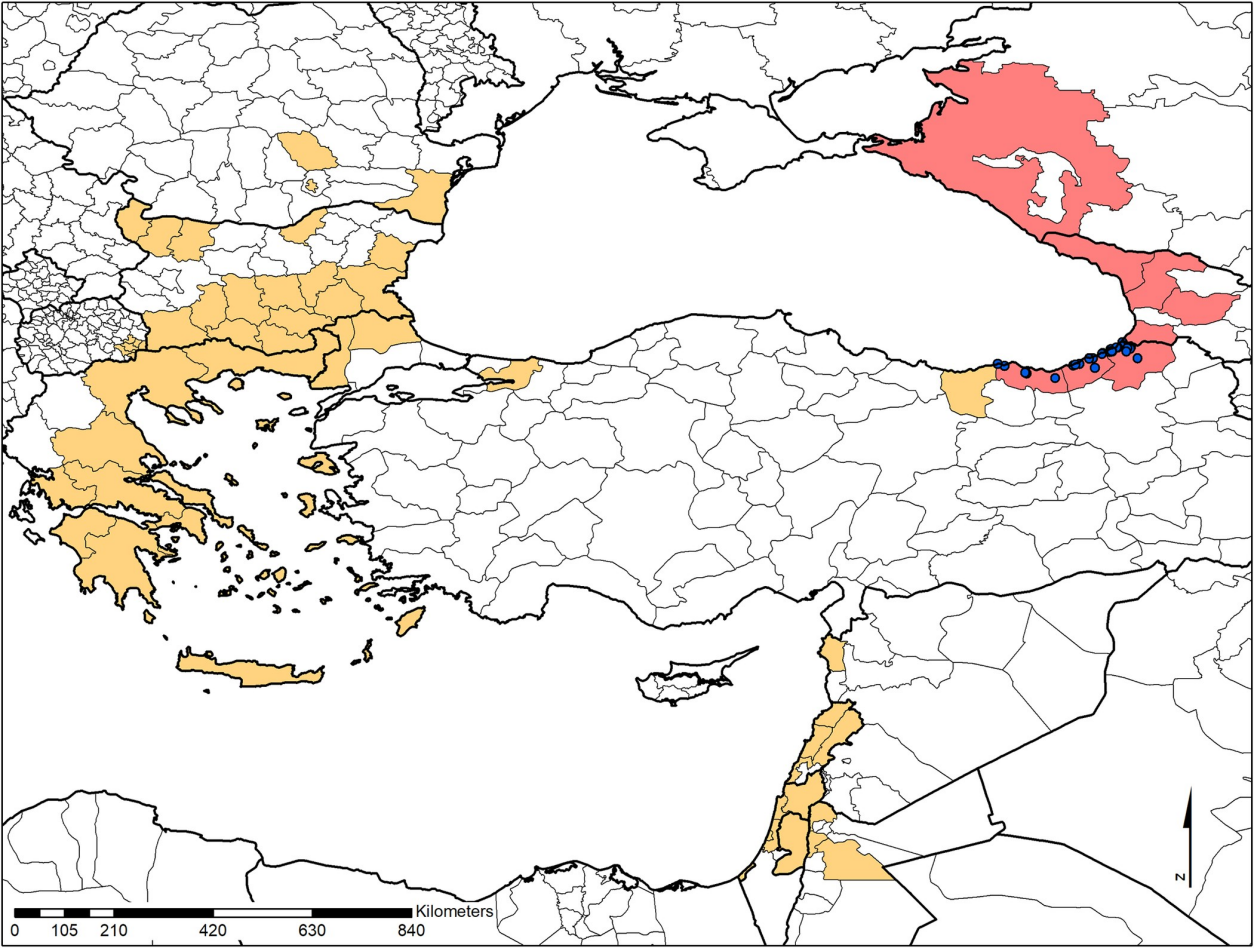
Map of the locations used for mosquito collection in the study. (Red: countries/territories with *Ae*. *aegypti* and *Ae*. *albopictus*; Orange: countries/territories with *Ae*. *albopictus*). Blue dots represent sampling locations. The baseline map has been prepared using Natural Earth raster + vector map data in the public domain (URL: www.naturalearthdata.com. Accessed: April 2019), which is freely available for personal, educational, and commercial use. Current information on *Aedes* species were obtained from the European Centre for Disease Prevention and Control websites (https://ecdc.europa.eu/en/publications-data/aedes-aegypti-current-known-distribution-june-2018; https://ecdc.europa.eu/en/publications-data/aedes-albopictus-current-known-distribution-june-2018; Accessed: December 2018).

Mosquito sampling was undertaken at 32 locations in Artvin, Rize and Trabzon provinces of the Black Sea region from June through October, in 2016 and 2017 (**Fig 1, S1 Table**). Members of the trained entomology team performed the samplings indoors and outdoors at urban sites using human landing catch method, as described previously [21]. Hepa Filter Mouth Aspirators and Prokopack Aspirator (John W. Hock Company, Gainesville, FL, USA) were employed specimen collection [22]. All collected mosquitoes were transferred on ice, killed by freezing and identified to species level using morphological keys [23,24]. Subsequently, the specimens were pooled according to the collection site, species and sex and stored at -80°C.

### Specimen processing and barcoding

Pooled mosquitoes were disrupted by vortexing following the addition of stainless steel beads (Qiagen, Hilden, Germany) and 500 microliters of Eagle’s minimal essential medium, supplemented with 5% fetal bovine serum, 1% L-glutamine and 1% penicillin-streptomycin. Subsequently, the pools were cleared by centrifugation at 4000 rpm for 4 minutes and the supernatants were collected. They were subjected to nucleic acid purificaton via High Pure Viral Nucleic Acid Kit (Roche Diagnostics, Mannheim, Germany), followed by reverse transcription for complementary DNA synthesis, using the RevertAid First Strand cDNA Synthesis Kit (Thermo Fisher Scientific, Hennigsdorf, Germany) by random hexamer priming, as directed by the manufacturer.

Mosquito pools with detectable virus sequences were further subjected to DNA barcoding for the confirmation of the species identification. For this purpose, a portion of the cytochrome c oxidase I (COI) gene was amplified and sequenced using LCO1490 and HCO2198 primers [25].

### Flavivirus and Zika Virus screening

A nested PCR assay with degenerated primers targeting the NS5 region of the flavivirus genome was used for screening in the mosquito pools [26]. The primers were reported to provide sensitive amplification of all insect-specific and major mosquito-borne flaviviruses including WNV, DENV, YFV, ZIKV, Saint Louis encephalitis virus and Usutu virus, strains with a detection limit of 40 TCID_50_ per reaction [26]. Assay optimization was undertaken using purified and serially-diluted nucleic acids from WNV NY99-4132 (standard) and T2 (local) isolates, grown on African green monkey (Vero) cells (ATCC- CCL81).

All pools were further tested individually for Zika virus, using the previously described real-time PCR assay targeting the envelope glycoprotein coding region [27]. The primers ZIKV1086-1162c and probe ZIKV1107 were used for the single step amplification via QuantiNova Pathogen+IC Kit (QIAgen, Hilden, Germany) in a Rotor-Gene 6000 instrument (Corbett Research, Australia). The assay was reported to have a detection threshold of 25 copies per reaction [23]. Zika virus strain UVE/ZIKV/1947/UG/MR766 RNA, obtained from the European Virus Archive, was used for assay optimization and as the positive template during screening.

### Alphavirus screening

Mosquito pools were screened for alphaviruses, using the nested PCR employing degenerate primers targeting the nsP4 region, as described previously [28]. The assay could amplify several medically-important alphaviruses including CHIKV, Sindbis, O'nyong nyong, western equine encephalitis, eastern equine encephalitis, Venezuelan equine encephalitis, Semliki forest, Ross river and Barmah forest viruses. The detection threshold of the assay was reported as 1–10 copies (cloned fragment) or 25 pfu (cell-grown virus) [24]. CHIKV strain LR2006-OPY1 cDNA, obtained from the European Virus Archive was used for optimization and as a positive template during screening.

### Rhabdovirus screening

We further screened the mosquito pools for the recently-characterized rhabdovirus, tentatively named Merida-like virus Turkey (MERDLVT) [29]. Two PCR assays, designed to amplify 481 and 160 base pairs from the L- and N- regions of the MERDLVT genome was used for screening the mosquito pools [29]. The assays were optimized using previously-collected positive mosquito pools.

Products of the screening assays were visualized under ultraviolet light following electrophoresis in 1.5–2.0% agarose gels, depending on the amplicon size. Precautions to prevent carry-over contamination were strictly followed and pre and post-PCR steps were performed in spatially-separated areas, with several non-template controls during each run.

### Virus isolation

Aliquots of the mosquito pools positive in the screening assays were inoculated onto semi-confluent monolayers of Vero (ATCC-CCL81) and *Ae*. *albopictus* (C6/36, ATCC-CRL1660) cells, following filtration through 0.22 micrometer sterile membrane filters (Merck Millipore, Darmstadt, Germany). The cells were incubated at 37°C and 28°C respectively, and monitored daily for cytopathic effects. Blind passages to fresh monolayers and testing for viruses via the screening assays were carried out weekly.

### Sanger and next generation sequencing (NGS)

Products of the mosquito COI barcoding and virus screening assays were cleaned up using PureLink PCR Purification Kit (Thermo Fisher Scientific, Hennigsdorf, Germany) and sequenced using forward-reverse primers of the particular assay and the BigDye Terminator v3.1 Cycle Sequencing Kit (Thermo Fisher Scientific) in an ABI PRISM 3500xL Dx genetic analyzer (Thermo Fisher Scientific).

Mosquito pools positive in the screening assays and with available aliquots were subjected to direct NGS. Following purification, the nucleic acids were reverse transcribed with random hexamer primers to double-stranded cDNA using SuperScript IV Reverse Transcriptase (Thermo Fisher Scientific, Hennigsdorf, Germany) and NEBNext mRNA Second Strand Synthesis Module (New England BioLabs, Frankfurt am Main, Germany). The Agilent 2100 Bioanalyzer (Agilent Technologies, Waldbronn, Germany) and Agencourt AMPure XP Reagent (Beckman Coulter Biosciences, Krefeld, Germany) were used for cleanup and estimation of yield and size distribution. Fragmentation, adaptor ligation and amplification were performed according to the manufacturer protocols using the NexteraXT DNA Library Preparation Kit (Illumina Inc., San Diego, CA, USA). Sequencing runs were performed on the Illumina MiSeq (Illumina Inc.) instrument in the paired end mode.

### Sequence data analysis

Raw sequences obtained from virus screening were handled using Geneious software v11.1.5 (Biomatters Ltd, Auckland, New Zealand). Trimmomatic [30] was used for adaptor removal, trimming for quality and length with a phred score of 33 and a minimum length of 30 base pairs (bp). Acquired reads were aligned to the RefSeq viral nucleotide and protein genome database using MALT (MEGAN alignment tool, v0.3.8) and MEGAN (Metagenome Analyzer, v. 6.12.3) [31,32]. Aligned reads were extracted and assembled into contigs using Velvet (v.1.2.10) [33]. The contigs were mapped to closely related virus strains, checked for heterogeneity via visual inspection and pairwise identity values using Geneious software v11.1.5 (Biomatters Ltd, Auckland, New Zealand).

BLASTn, BLASTn optimized for highly similar sequences (MEGABLAST) and BLASTp algorithms were employed for nucleotide and deduced amino acid similarity searches in the public databases, implemented in the NCBI website (www.ncbi.nlm.nih.gov/blast/) [34]. Nucleotide and putative amino acid alignments and pairwise sequence comparisons were generated via the CLUSTAL W program, implemented within Geneious software [35]. Nucleotide identity plots were generated by SimPlot version 3.5.1 [36]. Conserved protein domain and motif searches were performed using the web search tool (http://www.ncbi.nlm.nih.gov/structure/bwrpsb/bwrpsb.cgi) and MOTIF Search (http://www.genome.jp/tools/motif/) in the PFAM database [37,38]. Evolutionary history was inferred via the maximum-likelihood method based on the model estimated as the optimal substitution model individually for each alignment according to the Bayesian information criterion and conducted using MEGA6 [39].

## Results

A total of 791 specimens were studied (**Table 1**), which included 488 (61.7%) collected in 2016, and 303 (38.3%) individuals collected in 2017. *Aedes albopictus* was the most abundant mosquito species in all locations (n = 709; 89.6%), followed by *Ae*. *aegypti* (n = 62; 7.8%), and *Cx*. *pipiens* sensu lato (n = 20; 2.5%). Females comprised 91.4% (723 / 791) of the study cohort. Sampling sites in Artvin province provided 59.3% of the specimens (n = 496), followed by Rize (n = 192, 24.3%) and Trabzon (n = 130, 16.4%) provinces. *Aedes albopictus* and *Cx*. *pipiens* s.l. were detected in all provinces sampled, but *Ae*. *aegypti* was not detected in the collection sites in Trabzon province (**Table 1**).

**Table 1:** Overview of the field-collected mosquito specimens used for virus screening.

Species		Province						*Total*	
		Artvin		Rize		Trabzon			
		2016	2017	2016	2017	2016	2017		
*A*. *aegypti*	♀	7	0	45	4	0	0	56	62 (7.8%)
	♂	0	0	0	6	0	0	6	
*A*. *albopictus*	♀	305	114	39	63	92	35	648	709 (89.6%)
	♂	0	24	0	34	0	3	61	
*Cx*. *pipiens* s.l.	♀	0	19	0	0	0	0	19	20 (2.5%)
	♂	0	0	0	1	0	0	1	
*Total*		312	157	84	108	92	38	791	
		469 (59.3%)		192 (24.3%)		130 (16.4%)			

Mosquitoes were screened for viruses in 65 pools. Fifteen of these (23.1%) were reactive in at least one screening assay. Generic flavivirus assay was positive in all reactive pools, but the Zika virus specific and alphavirus PCRs were always negative. The only MERDLVT PCR positive was detected in a pool of DNA-barcode confirmed *Cx*. *pipiens* s.s., showing flavivirus reactivity. Virus isolation efforts were not successful in cell culture inoculation of the reactive pools. No cytopathic effect was observed in four consecutive blind passages and culture supernatants remained negative in flavivirus generic PCR. The mosquito species identification in reactive pools were confirmed via COI barcoding (**S1 Fig**).

### Flavivirus findings

**Fig 2:**
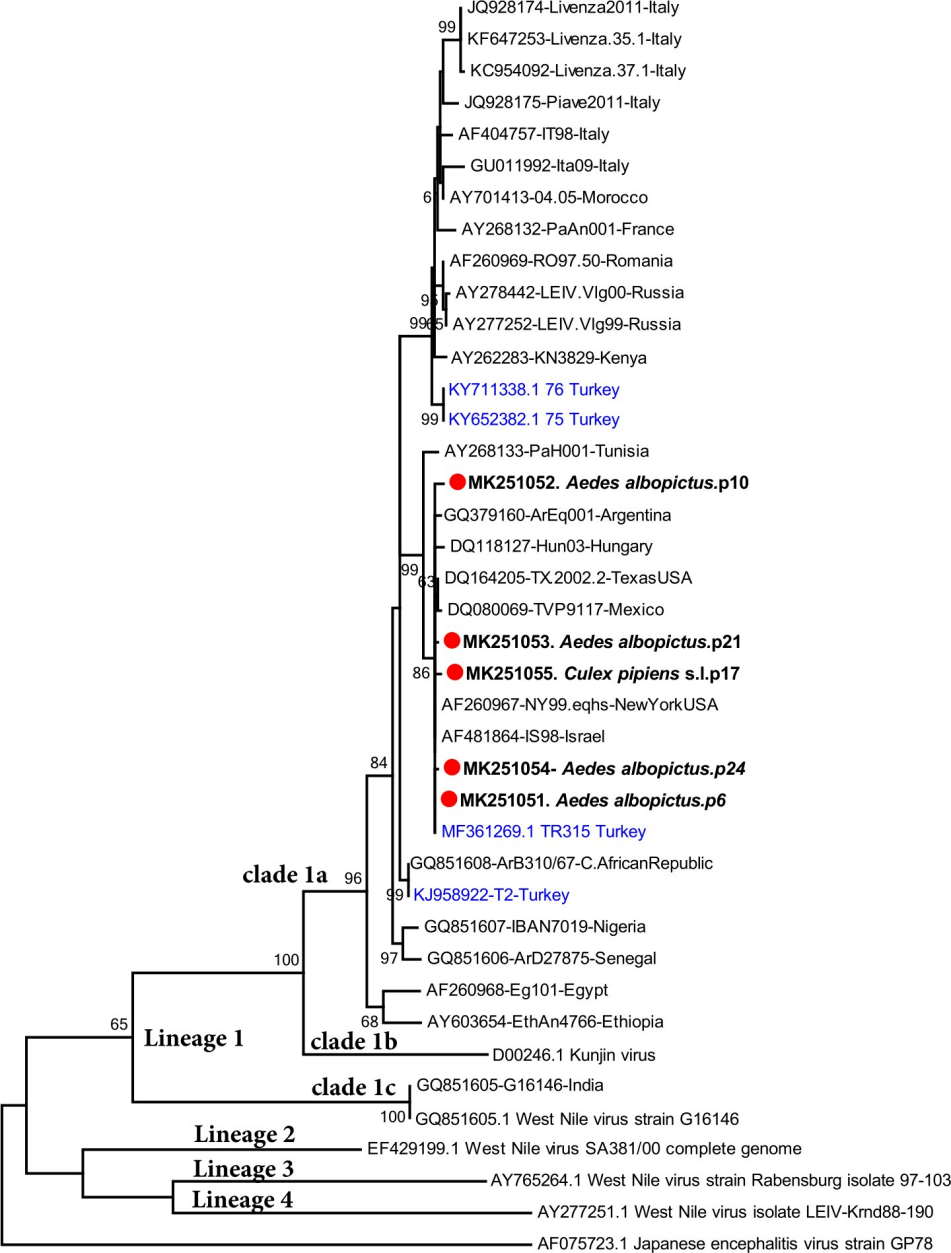
The maximum likelihood analysis of the partial West Nile virus NS5 sequences (777 nt). The tree is constructed using Maximum Likelihood method with the General Time Reversible (GTR) model, Gamma distributed with Invariant sites (G+I) for 1000 replications. The sequences characterized in this study are given in bold and indicated with a symbol, GenBank accession number, hosting mosquito species and pool code. Global virus strains are indicated by GenBank accession number, strain/isolate name and country of detection. Viruses previously characterized in Turkey are indicated with blue letters. Bootstrap values higher than 60 are provided. Japanese encephalitis virus strain GP78 is included as an outgroup.

The detected flavivirus was characterized via amplicon sequencing in PCR positive pools. WNV sequences were identified in 5 pools (5/65, 7.7%) comprising *Ae*. *albopictus* (4/5) and *Cx*. *pipiens* s.l. (1/5) mosquitoes (**Table 2**). Sequences of 777–1,000 base pairs (bp) were characterized. Pairwise comparisons showed diversity rates of 0.2–0.7% and 0.4–1.6% in the nucleotide and deduced amino acids, respectively. In the maximum likelihood tree, all sequences grouped within WNV lineage 1 clade 1a sequences, and formed a distinct cluster with viruses from the American Continent, Israel, Tunisia, Hungary and Aegean coast of Anatolia (Turkey) (**Fig 2**). Interestingly, WNV sequences of mosquito, equine and avian origins from Turkey grouped within different clusters in the lineage 1 clade 1a viruses.

**Table 2:** The mosquito pools with detectable virus sequences.

	Pool Code	Province	Site	Species–Pool content	Virus detected	Sequence characterized
1	KRD15	Artvin	A10	*Ae*. *albopictus* (21♀)	CFAV	Partial NS5 (MF361262)
2	KRD22	Artvin	A5	*Ae*. *albopictus* (10♀)	CFAV	Partial NS5 (MF361264)
3	KRD24	Rize	R1	*Ae*. *aegypti* (12♀)	CFAV	Partial NS5 (MF361265)
4	KRD30	Rize	R2	*Ae*. *aegypti* (10♀)	CFAV	Partial NS5 (MF361263)
5	KRD1	Artvin	A3	*Ae*. *albopictus* (13♀)	AEFV	Partial NS5 (MF361267)
6	KRD5	Rize	R2	*Ae*. *aegypti* (12♀)	AEFV	Partial NS5 (MF361268)
7	KRD32	Artvin	A3	*Ae*. *albopictus* (11♀)	AEFV	Near-complete genome (MK251047)
8	11	Artvin	A13	*Ae*. *albopictus* (4♀)	AEFV	Partial NS5 (MK251048)
9	20	Rize	R1	*Ae*. *albopictus* (21♀)	AEFV	Partial NS5 (MK251049)
10	26	Artvin	A15	*Ae*. *albopictus* (14♀)	AEFV	Partial NS5 (MK251050)
11	6	Artvin	A7	*Ae*. *albopictus* (2♀)	WNV	Partial NS5 (MK251051)
12	10	Artvin	A11	*Ae*. *albopictus*(33♀)	WNV	Partial NS5 (MK251052)
13	21	Rize	R6	*Ae*. *albopictus* (1♀)	WNV	Partial NS5 (MK251053)
14	24	Artvin	A15	*Ae*. *albopictus* (7♀)	WNV	Partial NS5 (MK251054)
15	17	Artvin	A1	*Cx*. *pipiens* s.s. (5♀)	WNV	Partial NS5 (MK251055)
					MERLVT	L region (MK251056)
						N region (S3 Fig)

**Fig 3:**
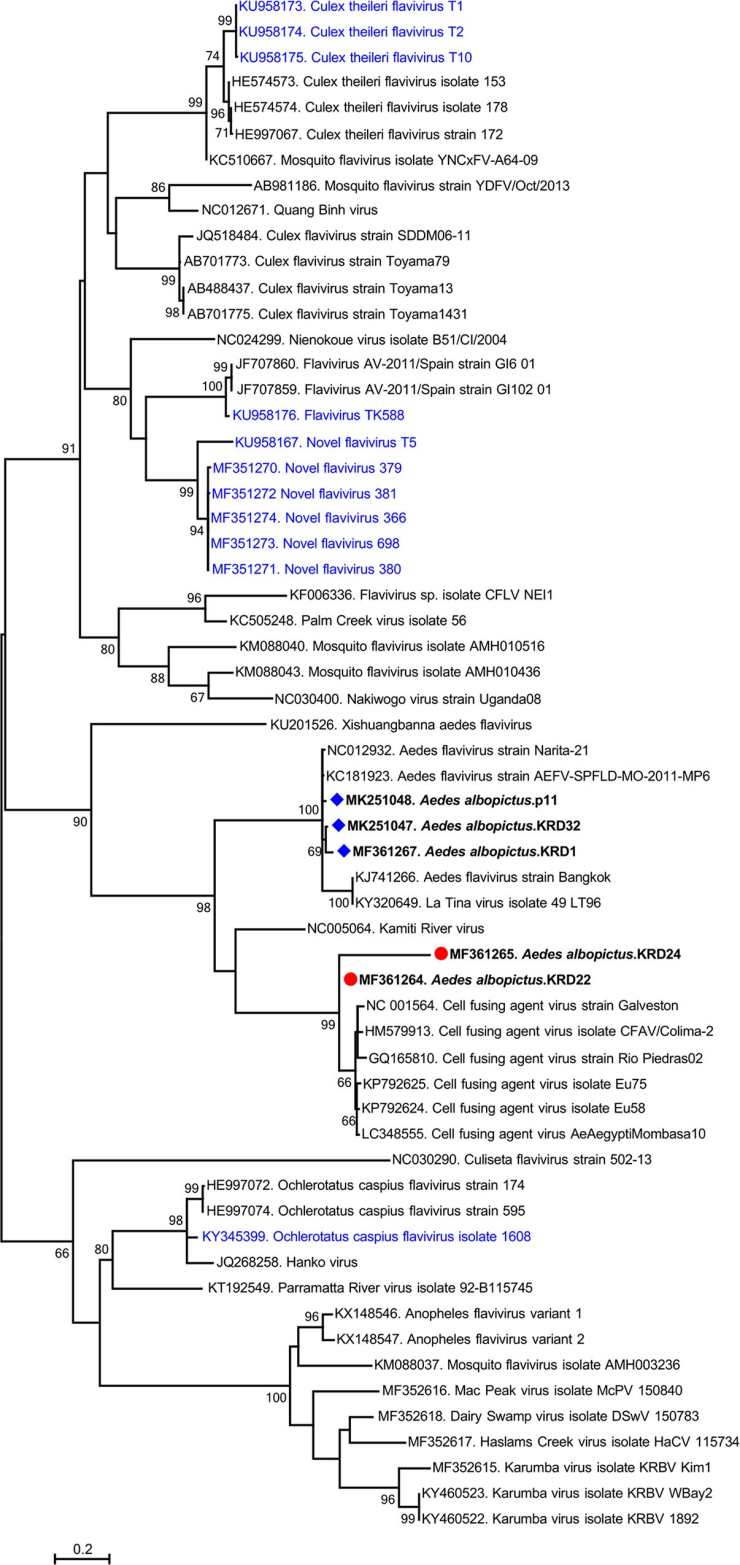
The maximum likelihood analysis of the partial flavivirus NS5 sequences (355 nt). The tree is constructed using Maximum Likelihood method with the General Time Reversible (GTR) model, Gamma distributed with Invariant sites (G+I) for 1000 replications. The sequences characterized in this study are given in bold and indicated with a symbol, GenBank accession number, hosting mosquito species and pool code. Global virus strains are indicated by GenBank accession number, virus and strain/isolate name. Viruses previously characterized in Turkey are indicated with blue letters. Bootstrap values higher than 60 are provided.

In 4 of the tested pools (6.2%) comprising 2 *Ae*. *albopictus and 2 Ae*. *aegypti* specimens, sequences identified as cell fusing agent virus (CFAV) were detected. The sequences were partially overlapping 283–695 bp segments, covering the flavivirus NS5 region amplicon. They displayed 86–97.8% identity to the CFAV strain Galveston (GenBank accession NC001564) in pairwise comparisons and formed a well-supported phylogenetic group with CFAV strains of various origins in the maximum likelihood tree (**Fig 3**).

Another flavivirus identified following amplicon sequencing was *Aedes* flavivirus (AEFV). AEFV was detected in a total of 6 pools (9.2%), 5 comprising *Ae*. *albopictus* (5/6) and 1 with *Ae*. *aegypti* (**Table 2**). The obtained sequences comprised 283–695 bp of partially overlapping segments of the target amplicon, with 0.9–8.8% intramural diversity in pairwise comparisons. Maximum likelihood analysis using the sequences from the pools KRD1, KRD32 and P11 revealed a separate clustering of these sequences with the closely-related AEFV strains, among other distinct insect flaviviruses, including CFAV (**Fig 3**).

**Fig 4:**
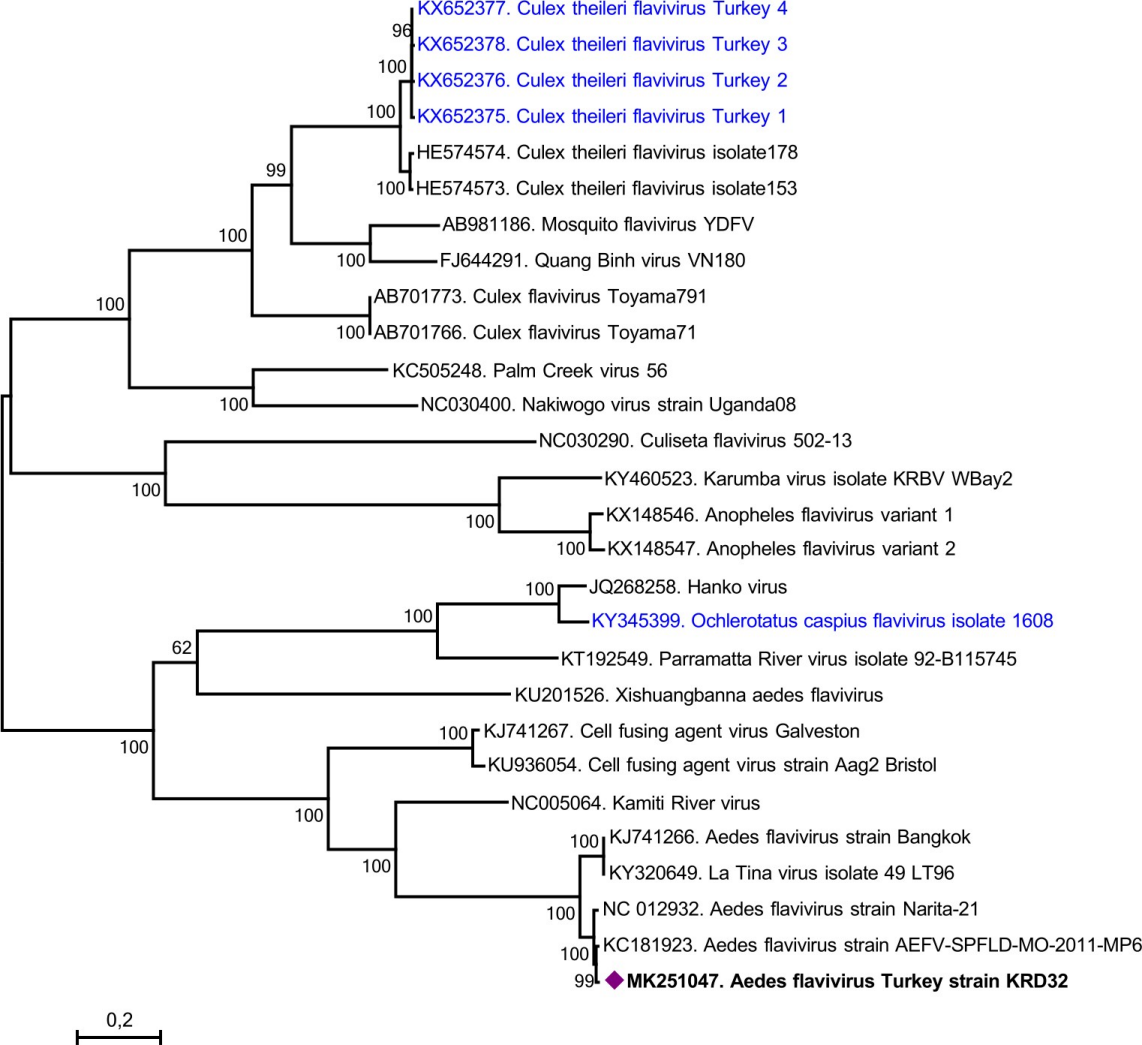
The maximum likelihood analysis of the near-complete polyprotein coding region (10766 nt) of selected insect-specific flaviviruses. The tree is constructed using Maximum Likelihood method with the General Time Reversible (GTR) model, Gamma distributed with Invariant sites (G+I) for 1000 replications. The sequence characterized in this study are given in bold and indicated with a symbol, GenBank accession number, virus and isolate/strain name. Global virus strains are indicated by GenBank accession number, virus and strain/isolate name. Viruses previously characterized in Turkey are indicated with blue letters. Bootstrap values higher than 60 are provided.

**Fig 5:**
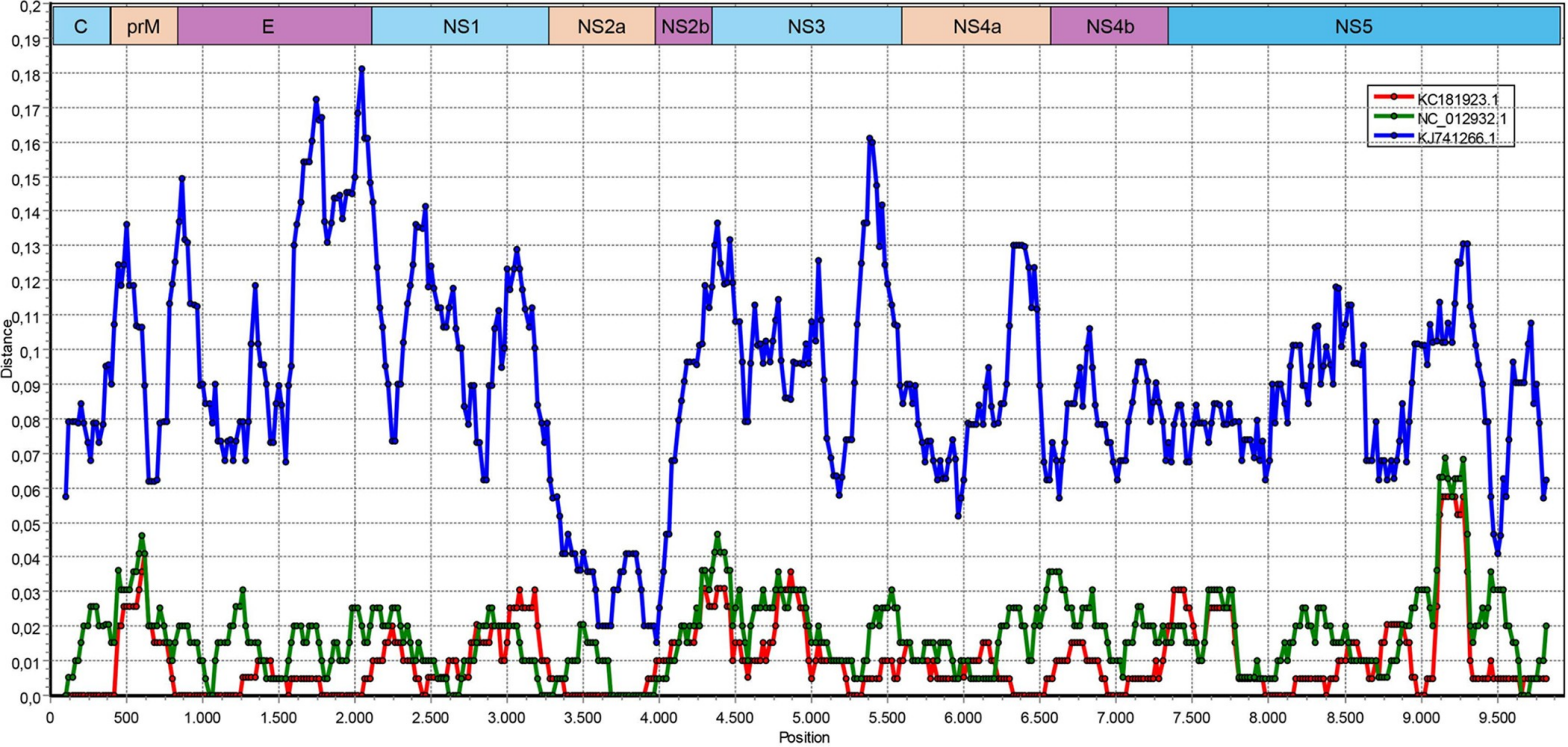
Plots of similarity of the near-complete polyprotein coding alignment (9931 nt) of AEFV-Turkey (MK251047), with individual functional units indicated (GapStrip: On, Reps: 1000, Kimura (2-parameter), T/t: 2.0). The curves indicate comparisons between the target and reference genomes (AEFV-Bangkok: KJ741266 AEFV-Narita21: NC012932, AEFV-SPFLD: KC181923). Each point plotted is the percent identity within a sliding window 200 bp wide centered on the position plotted, with a step size between points of 20 bp.

Despite lack of virus isolation in cell culture inoculation of flavivirus PCR positive pools, we could obtain near-complete polyprotein coding region of the AEFV via direct NGS in available aliquots in the mosquito pool, KRD32 (**Table 2**). The assembled sequence comprised 9,931 bp, with the deduced polyprotein of 3310 amino acids, that covered over 99% of the complete AEFV polyprotein. The sequence demonstrated pairwise diversity rates of 1–8.5% and 0.6–2.2% on the nucleotide and deduced amino acid levels, respectively; when compared to AEFV strains Narita-21 (GenBank accession AB488408), Bangkok (KJ741266), SPFLD-MO-2011-MP6 (KC181923) and the recently described La Tina virus isolate 49 (KY320649). Region-specific identities were further determined on the viral polyprotein, which revealed similarity rates of >90% on individual mature proteins (**Table 3**). Maximum likelihood analysis of the near-complete genome revealed a tree topology, comparable to the partial NS5 tree, with well-supported grouping of AEFV isolates (**Fig 4**). A genome-wide region specific nucleotide-based comparison is further provided in the SimPlot graph (**Fig 5**). The virus is provisionally named as AEFV-Turkey, as it represents the first near-complete AEFV genome reported from Asia minor. The insect-specific flavivirus ribosomal frameshifting site, that results in a longer overlapping ORF in the NS2A–NS2B regions [40], was observed as GGATTTT heptanucleotide motif, encompassing the nucleotides 3277–3283 in the AEFV-Turkey genome. Motifs of flavivirus envelope glycoprotein with the central/dimerisation domains (residues 306–478), flavivirus non-structural protein 1 (NS1) (879–1,052), NS3 serine protease (1,471–1,607), DEAD-like helicase domain (1,627–1,768), methyl transferase (2,475–2,663) and flavivirus RNA-dependent RNA polymerase (2,963–3,309) were identified in the deduced viral polyprotein. The envelope fusion peptide motif, involved in viral endosomal fusion and cellular entry [41], was located in 361–374. residues of the deduced viral polyprotein and characterized as NRGWGTGCFEWGLG.

**Table 3:** Functional organization of the AEFV-Turkey genome and comparison with related viruses. Similarity rates are given in percent.

	AEFV–Turkey		AEFV–Bangkok		AEFV—Narita21		AEFV—SPFLD		LaTina virus—LT96	
	*Position*	*Product*	*Nucleotide*	*Amino acid*	*Nucleotide*	*Amino acid*	*Nucleotide*	*Amino acid*	*Nucleotide*	*Amino acid*
C	1–376*	125 aa	0.933	0.968	0.986	0.976	1	1	0.933	0.968
prM-M	377–819	143 aa	0.907	0.944	0.975	0.986	0.981	0.972	0.907	0.944
E	820–2098	431 aa	0.892	0.974	0.985	0.995	0.997	1	0.892	0.974
NS1	2099–3265	389 aa	0.902	0.984	0.987	1	0.986	0.994	0.902	0.984
NS2a	3266–3967	234 aa	0.967	0.991	0.994	0.995	1	1	0.967	0.991
NS2b	3968–4342	125 aa	0.928	0.968	0.984	0.992	0.984	0.984	0.928	0.968
NS3	4343–5593	417 aa	0.902	0.966	0.977	0.98	0.985	0.988	0.902	0.966
NS4a	5594–6574	327 aa	0.918	0.978	0.984	0.993	0.993	0.996	0.918	0.978
NS4b	6575–7348	258 aa	0.925	0.988	0.981	0.992	0.993	1	0.925	0.988
NS5	7349–9931*	861 aa	0.919	0.986	0.979	0.99	0.986	0.995	0.919	0.986

**(**AEFVTurkey: MK251047, AEFVBangkok: KJ741266 AEFVNarita21: NC012932. AEFVSPFLD: KC181923, LaTina virus: KY320649)

* incomplete (aa: amino acid)

### Rhabdovirus findings

MERDLVT specific PCRs were positive in a single pool of molecularly confirmed *Cx*. *pipiens* s.s. (**Table 2**). Sequencing of the L-region amplicon provided a 470 bp segment, with 96.5–99.1% nucleotide and 91.6–92.3% amino acid identity to previously characterized strains. All MERDLVT sequences from Anatolia grouped as a distinct cluster in the maximum likelihood tree and shared common ancestor with Merida virus strains (**S2 Fig**). The N region amplicon further provided a 161 bp sequence, with 98.7% identity to the previously characterized MERDLVT genomes from isolates P431 and 139-1-21 (GenBank accessions MF882997 and KX951489) (**S3 Fig**). Alignment and pairwise comparisons revealed diversity rates up to 4.4% amongst sequences from specimens collected in various locations across Anatolia, Turkey (**S3 Fig**).

## Discussion

Targeted pathogen monitoring in arthropods is an important part of surveillance programs for the circulation of vector-borne agents and likely to predict probable disease emergence in susceptible human or animal populations [1]. The recent detection of invasive *Aedes* mosquitoes in Turkey, in parallel with the territorial expansion identified in several countries, have raised concerns about the reestablishment of these species as well as emergence of associated viruses [6,9]. This study was carried out to investigate the presence of the invasive *Aedes* species around the sites of previous detection and fill the current information gap on the circulation of viruses potentially spread by these mosquitoes. Specimen collection encompassed mosquito active seasons for two consecutive years, utilizing human landing catches, considered as a the most highly sensitive and effective approach for sampling anthropophilic *Aedes* species [21]. We detected *Ae*. *albopictus* and *Cx*. *pipiens* s.l. in all provinces targeted for surveillance and throughout the screening period (**Table 1**). However, *Ae*. *aegypti* was lacking in the Trabzon province, despite being identified regularly in the remaining provinces. *Aedes albopictus* was detected in high frequencies in all sampling sites, and comprised 89.6% of the study cohort overall. These findings indicate that these invasive *Aedes* are firmly established in the coastal Black Sea region of northeastern Turkey, at least in certain locations. It has been previously suggested that further spread of these species to the ports around the Black Sea via ships and ferries is probable, as well as dispersion via ground transportation into major cities of Turkey [9]. Therefore, continuous and integrated surveillance of invasive mosquitoes are imperative to monitor the spread of these species further into Asia Minor and to implement effective control strategies as become necessary.

We screened the field-collected mosquitoes for a diverse spectrum of viruses, including pathogenic flavi and alphaviruses mainly transmitted by *Aedes* mosquitoes, via generic or real-time PCR assays. WNV was noted as the prominent pathogenic agent, detected in 7.7% of the mosquito pools (**Table 2**). WNV circulates among various avian species and vector mosquitoes in nature. Humans and horses are exposed to the virus via infected mosquitoes and considered as dead-end hosts, due to the lack of prolonged and high-level viremia required to contribute to virus circulation [42]. WNV is widespread throughout Anatolia and the virus has been previously detected in field-collected mosquitoes including *Cx*. *pipiens* s.s., *Culex quinquefasciatus*, *Culex perexiguus*, *Aedes caspius* [14–16,43], in organ specimens from migratory birds [44], as well as in symptomatic humans and equine infections, occasionally presenting as outbreaks [12,13], However, most data on WNV circulation originated from Aegean, Mediterranean, Thrace and Central Anatolian locations with scarce information from the Black Sea region. We have identified WNV sequences in *Ae*. *albopictus* and *Cx*. *pipiens* pools collected from Artvin and Rize provinces in this study (**Table 2**), clearly indicating virus circulation in the region. We have previously evaluated mosquito specimens from Artvin province in 2013, comprising mostly *Culex* spp., without WNV detection [14]. However, WNV neutralizing antibodies were detected in 9.9% of the duck sera originating from the neighboring Kars province, suggesting prior virus exposure in the region [14]. It is known that different mosquito species possess highly variable potential to acquire and transmit WNV and *Culex* species are accepted as the primary global transmission vector [45,46]. WNV has been detected several other genera of mosquitoes including *Aedes*, *Anopheles*, *Coquillettidia*, *Culiseta*, *Mansonia*, *Mimomyia*, *Ochlerotatus*, *Psorophora*, and *Uranoteania*, that can serve as bridge vectors for transmission from birds to humans and equines [45,46]. *Aedes albopictus* mosquitoes are competent vectors for WNV, however their contribution to virus circulation in the field is considered as limited, due to their feeding preference for humans and variations in WNV transmission rates [47]. This may explain the lack of documented clinical cases from the screened provinces so far. Of note is the relatively hign WNV incidence in *A*. *albopictus* pools, which suggests considerable intensity of virus circulation in the sampling locations. Therefore, a detailed surveillance is required to better understand WNV epidemiology in the Black Sea region.

Five major WNV lineages have been described according to the genomic phylogenies, where lineage 1 is widely distributed throughout Africa, Asia and America [42]. However, other lineages also circulate in Europe where lineage 2 may cause human infections [48]. The partial WNV sequences characterized in mosquito pools in this study grouped phylogenetically with lineage 1 clade 1a sequences, which include the majority of the global lineage 1 strains as well as previously characterized sequences in Anatolia [11]. However, a significant WNV sequence diversity has also been documented in Turkey [16,42], which is represented in this study as differential clustering of mosquito, avian and equine sequences (**Fig 2**). This pronounced diversity, as well as occasional detection of lineage 2 strains [49], probably results from independent virus introductions and dispersion via migrating birds throughout Anatolia and Thrace [43].

Besides pathogenic flaviviruses, our screening provided information on insect-specific flaviviruses (ISFs) in mosquitoes of the Black Sea region of Anatolia. Forming a major phylogenetic group of *Flaviviridae*, the ISFs do not seem to infect vertebrates and replicate exclusively in mosquito derived cell lines [40]. ISFs demonstrate a widespread geographic distribution and have been detected in a wide range of mosquito species, including those that act as virus vectors. Therefore, they share identical ecologic niches with vector-borne pathogens and frequently co-circulate in given geographical areas [40]. We have identified two distinct ISFs, CFAV and AEFV, in this study. They could be detected in several sampling sites during consecutive years in *Ae*. *albopictus* and *Ae*. *aegypti* mosquitoes (**Table 2**), suggesting dissemineated circulation in the region. CFAV was the first ISF to be discovered, initially isolated from an infected *Ae*. *aegypti* cell line [50]. It has subsequently been detected in several *Aedes* and *Culex* spp. mosquitoes from locations in Asia, Africa and the American continent [40], and recently reported from Brasil [51]. AEFV is another ubiquituous ISF, previously also detected in mosquitoes from Italy and United States, following its initial characterization in Japan [52–56]. Mostly identified in *Ae*. *albopictus* and *Aedes flavopictus* mosquitoes, AEFV was also present in *Cx*. *pipiens* mosquitoes from Italy, suggesting transspecies infections similar to CFAV [55]. Both viruses were also isolated from laboratory colonies established from mosquitoes collected in Thailand and the United States [57]. In our study, the local AEFV strain provisionally named as “AEFV-Turkey”, was detected in *Ae*. *aegypti* as well as in *Ae*. *albopictus* mosquitoes (**Table 2**). The near complete genome of AEFV-Turkey revealed significant sequence similarities, identical organization with specific regions and functional markers with AEFVs (**Table 3, Fig 5**). We have previously isolated and characterized several ISFs in Anatolia and Thrace, including *Culex theileri* flavivirus Turkey [58], *Ochlerotatus caspius* flavivirus Turkey [16], *Anopheles* flavivirus and evidence for novel ISFs [43]. Therefore, AEFV-Turkey becomes the latest addition to the list of ISFs known to circulate in the Anatolian mosquito fauna. Of particular interest is the probable interaction of ISFs with pathogenic flaviviruses, resulting in alterations in vector infection efficiency or transmission dynamics [40]. However, currently available information is insufficient for well-supported conclusions and detailed screening is likely to provide epidemiological data required for a better understanding of the ISF-pathogen interactions occuring in nature.

We have further detected MERDLVT in a pool of *Cx*. *pipiens* s.s. mosquitoes, also positive for WNV (**Table 2**). MERDLVT and closely related MERDV are putative members of the family *Rhabdoviridae*, identified using NGS without isolation in field-collected mosquitoes from Mexico and Turkey, without succesful isolation of a viable strain [29,59]. MERDLVT has previously been detected in *Cx*. *pipiens* mosquitoes from several locations in Mediterranean and Aegean Anatolia as well as from Thrace [29,43]. The detection of MERDLVT within a relatively small *Culex* cohort indirectly suggests prominent virus circulation in the Black Sea region. MERDLVT has so far been observed exclusively in *Culex* mosquitoes [29,43]. However, mosquitoes with MERDV infection are not limited to *Culex* genera, and partial viral sequences were detected in *Aedes taeniorhynchus* and *Aedes trivittatus* mosquitoes among seven mosquitoes species in Mexico [59]. The MERDLVT sequences are generally well-conserved, as observed in the amplified sections of the viral L and N genes in this study, and phylogenetically-related to the previously identified sequences (**Fig 4**). We have previously documented the cocirculation of flaviviruses and MERDLVT, which is also observed in this study [43]. Similar to ISFs, the impact of MERDLVT or MERDV on vector survival and pathogen transmission remains currently unexplored and requires further investigation.

In conclusion, we have identified ongoing activity of invasive *Aedes* mosquitoes in Black Sea region of Anatolia. WNV circulation is documented for the first time in potential mosquito vectors in the region. No evidence of recently-emergent Zika, Chikungunya or other pathogenic flavi/alphavirus was observed. Insect-associated flavi and rhabdoviruses were detected, with near-complete genome of AEFV, reported initially from Anatolia.

## Supporting information

S1 Table Locations used for mosquito sampling. (XLS)

S1 Fig The neighbor joining analysis of the mosquito COI sequences. The tree is constructed using maximum composite likelihood method for 1000 replications. The sequences obtained in mosquito pools in this study are given in bold and indicated with species and pool code. Reference sequences are indicated by GenBank accession number, species and country of collection. Bootstrap values higher than 60 are provided.
(PDF)

S2 Fig The maximum likelihood analysis of the MERDLVT partial L region. The tree is constructed using Maximum Likelihood method with the General Time Reversible (GTR) model, Gamma distributed with Invariant sites (G+I) for 1000 replications. The sequence characterized in this study are given in bold, indicated with a symbol and the host mosquito species. Global virus strains are indicated by GenBank accession number, virus and strain/isolate name. Bootstrap values higher than 60 are provided.
(PDF)

S3 Fig Alignment of the MERDLVT partial N region sequences. A: with complete genomes, B: with Merida virus and local strains.
(PDF)
