# Supplementary figures and images for: Arboviral screening of invasive Aedes species in northeastern Turkey: West Nile virus circulation and detection of insect-only viruses
Source: PLoS Negl Trop Dis. 2019 May 6;13(5):e0007334. doi: 10.1371/journal.pntd.0007334 (PMC6522068; doi:10.1371/journal.pntd.0007334)

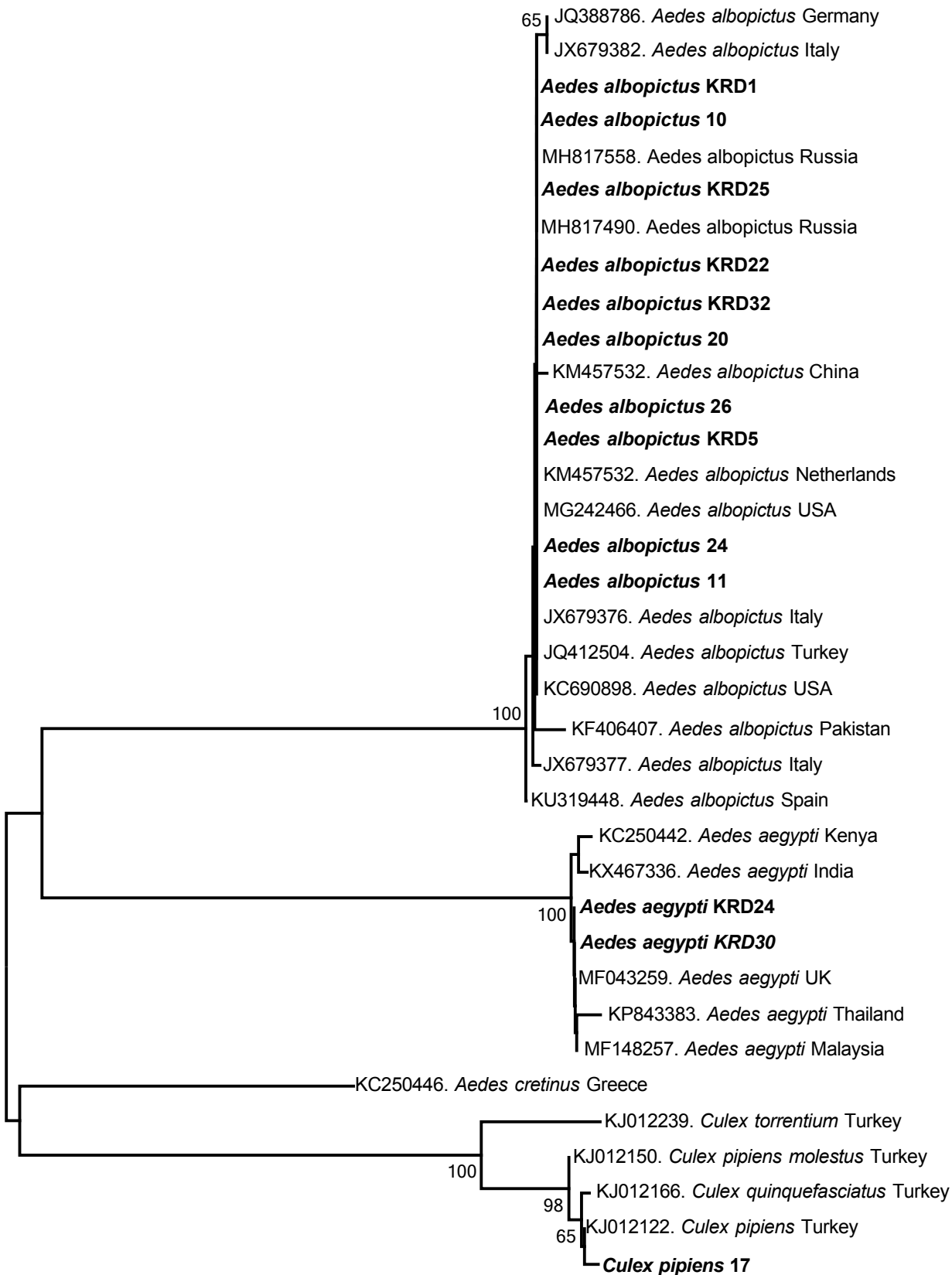

0.02

Supplement: S1 Fig — The tree is constructed using maximum composite likelihood method for 1000 replications. The sequences obtained in mosquito pools in this study are given in bold and indicated with species and pool code. Reference sequences are indicated by GenBank accession number, species and country of collection. Bootstrap values higher than 60 are provided. (PDF) [file pntd.0007334.s002.pdf]

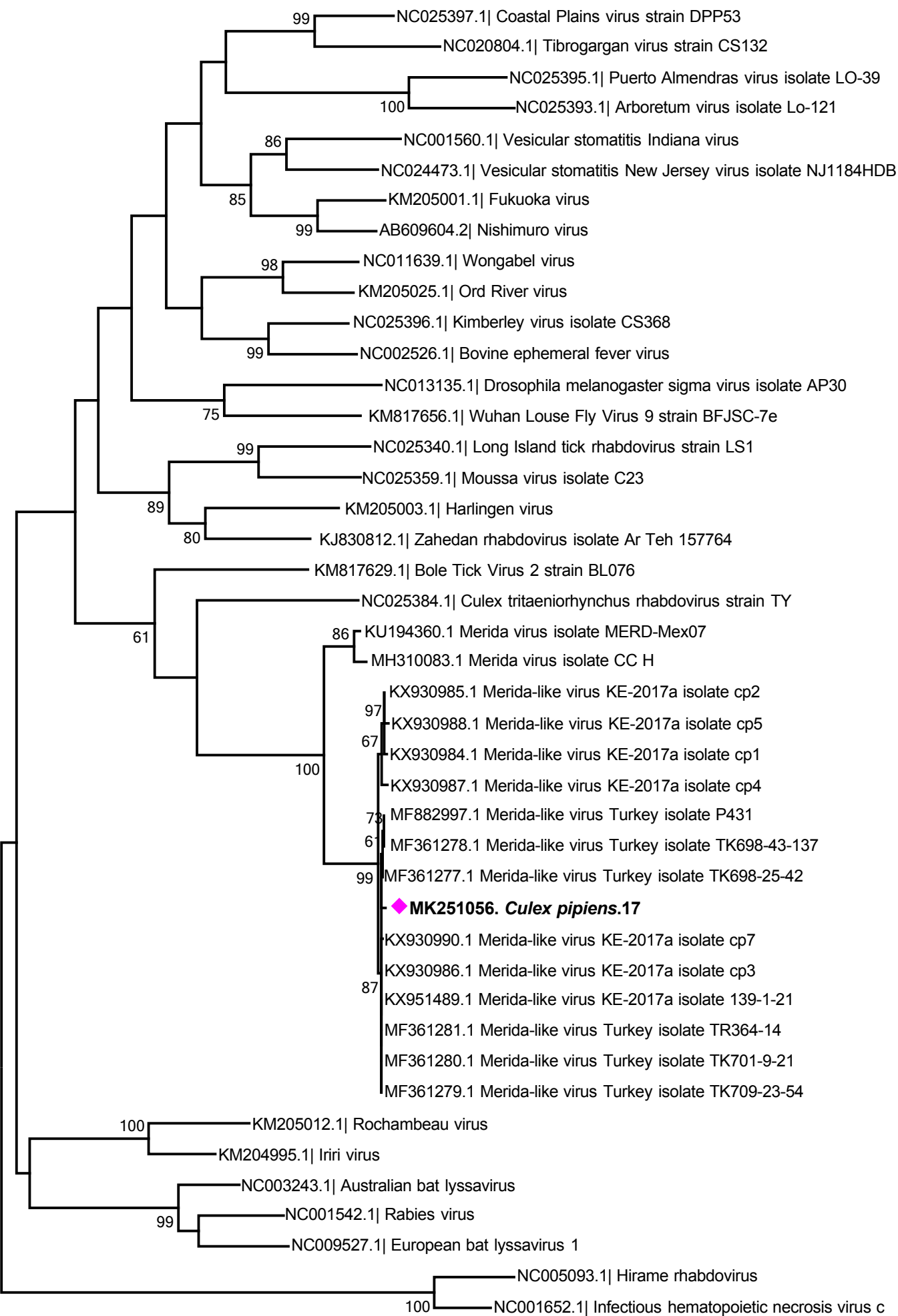

Supplement: S2 Fig — The tree is constructed using Maximum Likelihood method with the General Time Reversible (GTR) model, Gamma distributed with Invariant sites (G+I) for 1000 replications. The sequence characterized in this study are given in bold, indicated with a symbol and the host mosquito species. Global virus strains are indicated by GenBank accession number, virus and strain/isolate name. Bootstrap values higher than 60 are provided. (PDF) [file pntd.0007334.s003.pdf]
