# Supplementary material for: Arboviral screening of invasive Aedes species in northeastern Turkey: West Nile virus circulation and detection of insect-only viruses
Source: PLoS Negl Trop Dis. 2019 May 6;13(5):e0007334. doi: 10.1371/journal.pntd.0007334 (PMC6522068; doi:10.1371/journal.pntd.0007334)
Supplement: S3 Fig — A: with complete genomes, B: with Merida virus and local strains. (PDF) [file pntd.0007334.s004.pdf]

A

```
                    10        20        30        40        50        60        70        80        90        100
           ....|....|....|....|....|....|....|....|....|....|....|....|....|....|....|....|....|....|....|....|
P431       CGCTCCTGATATGGCTACTACCGCTACCTTGATTCCATCTAACCCTTACGAGGGACTTGACGGAGATGTAGATTGCACGAGCTTCAGCGATCTCGCGGCC
139-1-21   ....................................................................................................
17cp       ....................................................................................................

                   110       120       130       140       150       160
           ....|....|....|....|....|....|....|....|....|....|....|....|.
P431       GAAAACGGGCAACCGTCGACATGGGAGGTAGTGGAATCCACTCACATGGGTGGGACCGAGA
139-1-21   .............................................................
17cp       .............................G......C........................
```

B

```
                  10        20        30        40        50        60        70        80        90       100       110
         ....|....|....|....|....|....|....|....|....|....|....|....|....|....|....|....|....|....|....|....|....|....|....

MERDV    CCACTTTGATTCCTTCTAACCCTTACGAGGGACTTGAAGGAGATGTGGATCACACAGGCTTCAGTGATCTGTCGGTCAAAACCGGGGAAACGTCGACATGGGGTGTCGTAGAGT
MERDLVT  .T..C........A.......................C........A...TG...GA.......C.....CG...C.G...A....C..C............AG..A..G..A.
cp1      .T..C........A.......................C........A...TG...GA......TC.....TG.A.C.G...A....C..C............AG..G..G..AC
cp2      .G..C........A.......................C........A...TG...G.......TC.....TG.A.C.G...A....C..C............AG..G..G..AC
cp3      .T..C........A.......................C........A...TG...GA.......C.....CG...C.G...A...AC..C............AG..A..G..AC
cp4      .T..C........A.......................C........A...TG...GA.......C.....TG...CAG....A...C..C............AA..G..G..AC
cp5      .T..C........A.......................C........A...TG...GA......TC.....TG.A.C.G...A....C..C............AG..A..G..AC
cp6      .T..C........A.......................C........A...TG...GA......TC.....TG.A.C.G...A....C..C............AG..A..G..AC
cp7      .T..C........A....................C..C........A...TG...GA.......C.....CG...C.G...A....C..C............AG..A..G..AC
cp8      .T..C........A.......................C........A...TG...GA......TC.....TG.A.C.G...A....C..C............AG..G..G..AC
P254     .T..C........A.......................C........A...TG...GA.......C.....CG...C.G...A....C..C............AG..A..G..AC
P431     .T..C........A.......................C........A...TG...GA.......C.....CG...C.G...A....C..C............AG..A..G..A.
P709     .T..C........A.......................C........A...TG...GA.......C.....CG...C.G...A....C..C............AG..A..G..AC
P701     .T..C........A.......................C........A...TG...GA.......C.....CG...C.G...A....C..C............AG..A..G..AC
P364     .T..C........A.......................C........A...TG...GA.......C.....CG...C.G...A....C..C............AG..A..G..AC
17Cp     .T..C........A.......................C........A...TG...GA.......C.....CG...C.G...A....C..C............AG..G..G..AC
```
